# Address

**Published:** 1876-05

**Authors:** 


### ADDRESS.

BY DR. FITCH.

*Read before the Michigan State Dental Society.*

GENTLEMEN:

In retiring from the honorable position to which your partiality and kind consideration called me, I desire to express my hearty thanks, not only for the honor conferred, but also for the many kindly courtesies extended to me as President of this association.

I shall attempt in a brief and practical manner to speak of the duties which we, as an association, owe to the profession of which we are members; to the State of which we are citizens; to the individuals with whom we are brought in professional relationship; and last, but not least, to that department of the University which has been lately established in the interest of the public, and for the elevation of our profession, viz: the DENTAL COLLEGE.

The duties which we owe to the dental profession involve a proper and adequate preparation for the laborious and delicate duties which we are called upon to perform; honorable relations and manly conduct to our brethren, and a life molded and fashioned by a correct deportment, and by the exhibition of those principles of virtue and uprightness which should always mark the professional gentleman. We have passed that period in the history of our State, when the work of the dentist can be legitimately performed by the mere mechanic. Preparation is not only desirable but absolutely necessary. Not only is mechanical skill required, not only must a dentist possess tact and judgment and delicacy of manipulation, but he must also have a very respectable knowledge of anatomy, physiology, chemistry, materia medica, and the great principles underlying the whole range of knowledge relating to pathological research and investigation.

I simply call attention to what will be admitted by every intelligent dentist, and leave the subject as a text for thought, for not only such persons as contemplate entering upon the study of dentistry, but also as an incentive to laborius study, to such, particularly the younger members of the profession, who may not have had the advantages here indicated.

Our relations with members of the profession should be honorable and courteous. Unkind criticisms of the work performed by others, that species of advertising which claims marked superiority over our competitors, underbidding our brethren, or in any manner engaging in a dishonorable strife for the purpose of attracting patronage, will never be practiced by a man who is true to the profession, which is being more and more recognised, year by year, as one of the learned professions.

The profession has a right to demand of its members correct deportment and virtuous conduct in the every day walks of life, as well as the exercise of due courtesy to professional brethren. A life of sobriety, purity and respectability, not only sheds a lusture on the man who leads it, but also on the profession of which he is a member.

The intelligence of our people—the citizens of Michigan—is not only recognised in every State in the Union, but is spoken of with admiration in other countries. The various professions, divinity, law and medicine, may point with pride to their many distinguished members; the mechanic and the farmer of the State are to a large extent reading and cultivated men; the great lumber and other industrial interests in the northern portion of our territory are represented by men of not only remarkable force of character but in many instances of scholarly attainments. The profession of dentistry having to do largely with this intelligence can not afford to occupy a backward or doubtful position. Our duty is plain. We must support the various educational institutions which bless our land; aid in the maintenance of social virtues and of good government; range ourselves on the side of every movement and every effort calculated to elevate the people, promote the general interests of all classes and thus ennoble mankind.

The dentist is brought into intimate and confidential relations with his patients, To persons asking his advice and submitting to his treatment, he should be frank, truthful and gentlemanly. Persons of delicate health, easily susceptible to every influence, whether kind or unkind, which may surround them; persons whose nervous system has been shattered by disease and pain; in a word every condition of humanity in all its suffering, anguish and pain, seek his advice and avail themselves of his professional skill. To his patients he should be kind, firm, gentle, patient, truthful and judicious; all the tact of which he is possessed will often be brought into requsition; that tact should be cultivated and developed, and the patient should receive an elevated idea of the profession from the character, conduct and ability of the individual members. Our association should include in its list of members every dentist in the State whose moral qualifications and professional acquirements entitle him to recognition, and those who are not thus qualified should be induced to seek some other employment, he can be of no possible use to the dental profession.

Other states are procuring legislative enactments, defining the qualifications and regulating the practice of dentistry. If we would not have this State the receptacle for those who are prohibited from practicing in other states, it will be necessary for us to take some action to secure like legislation.

I would suggest that this matter be referred to a committee to be appointed by this association, with instructions to present the matter to the next Legislature for its action.

A new and important duty devolves upon us, growing out of the fact that a Dental College has been established in connection with and as a part of the University of Michigan. The Legislature generously and with a proper regard to the development of dental science, made an appropriation, scanty it is true, but nevertheless sufficient, with care and economy, to begin the work of dental education. The Regents, promptly and in good faith, began the work of laying the foundation, consulting freely with dentists from all parts of the State, as to the best plans to be devised to secure its success. Persons of eminence in the profession were chosen to fill the chairs. The work has been commenced, and we are abundantly satisfied with its beginning. Its success will depend largely upon the exertions of the Profession.

When we asked the Legislature to make the appropriation to found this college, we pledged it our support. Let us then give to its distinguished head, and also to his co-laborers our united confidence; assuring the people who are taxed to maintain this interest, of its early and permanent success. When we consider the plan of the college, and the advantages which accrue to it. From its connection with the medical department, and indeed with the University, we can conscientiously recommend it to every dental student who desires to prepare himself for scientific work, confident that we have an institution of professional learning second to none in the country in character and usefulness.

